# Assessing the survival of exogenous plant microRNA in mice

**DOI:** 10.1002/fsn3.113

**Published:** 2014-05-15

**Authors:** GaoFeng Liang, YanLiang Zhu, Bo Sun, YouHua Shao, AiHua Jing, JunHua Wang, ZhongDang Xiao

**Affiliations:** 1 State Key Laboratory of Bioelectronics, School of Biological Science and Medical Engineering, Southeast UniversityNanjing, 210096, China; 2 School of Medical Technology and Engineering, Henan University of Science and TechnologyLuo Yang 471003, Henan, China

**Keywords:** Blood stream, cross-kingdom, gastrointestinal, miR-172, organs

## Abstract

MicroRNAs (miRNAs), a class of small RNAs, are important molecules that influence several developmental processes and regulate RNA interference (RNAi), and are abundant in animals, plants, and plant tissues that are traditionally consumed in the diet. The survival of plant small RNAs from the diet in animals, however, remains unclear, and the persistence of miRNAs from dietary plants in the animal gastrointestinal (GI) tract is still under debate. In this study, ICR mice were fed plant total RNAs in quantities of 10–50 *μ*g, extracted from *Brassica oleracea*. Serum, feces, and various tissues were collected from the mice after RNA consumption and analyzed for several miRNAs. Exogenous plant miRNAs were present in the sera, feces, and tissues of animals and these exogenous plant miRNAs were primarily acquired orally. MiR-172, the most highly enriched exogenous plant miRNA in *B. oleracea*, was found in the stomach, intestine, serum, and feces of mice that were fed plant RNA extracts including miR-172. The amount of miR-172 that survived passage through the GI tract varied among individuals, with a maximum of 4.5% recovered at the stomach of one individual, and had a range of 0.05–4.5% in different organs. Furthermore, miR-172 was detected in the blood, spleen, liver, and kidney of mice.

## Introduction

MicroRNAs (miRNAs) are a class of small RNAs (19–25 nucleotides (nt) long) which have posttranscriptional activity in animals and plants (Lee and Ambros 2001; Carrington and Ambros 2003; Bartel 2007). They are generated from endogenous hairpin-shaped miRNA precursors, transcribed from nonprotein-coding genes. The precursors form self-complementary fold back structures and are processed by the RNase III-like nuclease Dicer (animals) or Dicer-like 1 protein (plants) (Denli and Hannon 2003). The wide range of functions of miRNAs is largely due to the complex relationship with their target genes. In general, target genes that are completely complementary to the miRNA will be degraded through an RNA-interference mechanism, whereas targets with partial complementary sequences at their 3′-UTR are subjected to translation inhibition and to a lesser extent mRNA degradation. In plants, a near-perfect complementarity between miRNAs and protein coding genes is readily seen, whereas in mammals partial complementarity is more common (Doench and Sharp 2004; Bagga et al. 2005; Lim et al. 2005; Pillai et al. 2005).

Increasing evidence has demonstrated that miRNAs regulate several functional genes involved in a wide range of developmental processes, including cell proliferation, differentiation, apoptosis, cell growth, and cell death in both plants and mammals (Carrington and Ambros 2003; Ambros 2004; Bartel 2007; Alexander et al. 2013; Lefort et al. 2013). Since the first miRNA was discovered in *Caenorhabditis elegans* in 1993 (Lee et al. 1993), thousands of miRNAs have been found in all plants, animals, and in other eukaryotes. Among other roles, miRNAs appear to regulate the development of leaves and flowers in plants (Mallory et al. 2002; Reinhart et al. 2002), early larval transitions, and cell growth and death in animals (Chatterjee and Grosshans 2009; Van Wynsberghe et al. 2011; Huang et al. 2012). To the best of our knowledge, however, there are no published studies showing that identical miRNAs exist between animal and plant kingdoms.

In this report, we investigated the survival of exogenous RNA in the digestive system, blood, and several organs of mice after being fed RNAs extracted from *Brassica oleracea*. Upon gavage with exogenous plant total RNAs, plant-specific small RNAs in the animals were found. When plant RNAs were added directly to food pellets, plant miRNA fragments were also detected in the feces, blood, gastrointestinal (GI) tract, and organs.

Although the functional effect of those plant-specific miRNAs in mammals is under debate, first their ability to survive the mammalian GT tract and enter blood and organs needs to be verified. A plant specific miRNA, miR-172, was identified in the feces by polymerase chain reaction (PCR) and validated by TaqMan® assay. These results show that exogenous plant miRNA can survive in the murine GI tract, enter peripheral blood, and continue to other organs.

## Materials and Methods

### Feeding of animals

All animal experiments were performed in accordance with permission from the ethics committee of Southeast University, China. All animal experiments were performed using ICR strain mice on a 12-h light/dark cycle in a pathogen-free animal feeding facility at Southeast University. Male and female ICR mice between the ages of 2–4 months were gavage fed total RNAs extracted from *B. oleracea* (see below) in DEPC-treated water in quantities of 10 to 50 *μ*g in a total volume of 500 *μ*L. The solution was administered from a pipette tip directly into the oral cavity. In some of the feeding experiments, the same amount of RNAs solution was added to the food offered as diet to the animals. As a control, prior to feeding, a fecal sample from each experimental animal was examined to confirm the absence of miRNA sequences with homology to miR-172 (data not shown).

### Preparation of plant RNA and miRNA screening

Total RNA was extracted from *B. oleracea*, *Brassica rapa*, rice, corn, *Arabidopsis thaliana*, and *Solanum lycopersicum* using Trizol® (Invitrogen, Carlsbad, CA) according to the manufacturer's protocol. The concentration of total RNA was quantified by the absorbance at 260 and 280 nm. The total RNA was electrophoresed in 1.6%/Tris-Boric acid-EDTA buffer agarose gel to visualize small RNAs. A stem-loop reverse transcription polymerase chain reaction (RT-PCR) assay (Chen et al. 2005; Tang et al. 2006) was adapted to screen-specific mature miRNA expression in plants and fecal samples of control mice. For the remaining feeding experiments, total RNA extract from *B. oleracea* was used.

### Degradation of RNA in blood, and fecal suspensions

To determine the degradation of plant small RNA in vitro, total RNA (5 *μ*g) was incubated at 37°C with freshly drawn serum or fecal suspension in DEPC-treated water from control mice. At various times after starting incubation (2, 4, 10, 18, 24, and 36 h), RNA samples were prepared, purified (see below), and analyzed for the presence of small RNA sequences by electrophoresis.

### Tissue, blood, and fecal sample collection

At various times (2, 4, 6, 9, 12, 24, and 72 h) after feeding the exogenous RNA solution to mice, fecal samples were collected. Mice were then anesthetized with ether, the abdominal fur was thoroughly washed with ethanol, and one set of surgical instruments was used to open the peritoneal and thoracic cavities by cutting the diaphragm. Finally, the still palpitating heart was punctured with a disposable 0.9-mm gauge needle, and blood (about 0.5–1 mL) was drawn with a syringe. For facilitation, in some of the experiments, blood was taken from the fundus of eyes in the mice. Thereafter, the contents of the stomach, intestines, liver, spleen, and kidneys were removed for analyzing. Control analyses on abdominal fur washing fluids performed by PCR methods revealed no evidence for detectable contamination.

### Isolation and purification of total RNA from contents of the stomach, intestines, and fecal samples

The contents of the stomach, intestines, and feces were dispersed in 0.5 ml DEPC-treated PBS, respectively. The suspensions were incubated at 37°C for 15 min, and subsequently nucleic acids were extracted according to the methods described above. These extracts were subsequently treated with 10 U of DNase per mL at 37°C for 30 min. Prior to use, the DNase was heated to 85°C for 10 min to destroy contaminating DNase.

### Extraction of total RNA from liver, spleen, kidney, and blood

Total RNA was extracted using Trizol® from the liver, spleen, kidney, and blood according to the standard protocol. In some of the experiments, the blood cells were pelleted by low-speed centrifugation, and RNA was extracted separately from the cell pellet and serum by the SDS-proteinase K-phenol/chloroform procedure. Next, DNase treatment was performed to eliminate residual DNA contamination. Finally, after a second acid phenol:chloroform extraction, the RNA was resuspended in DEPC-treated water.

### Detection of miR-172 in fecal extracts, tissues, and blood samples by conventional RT-PCR and real-time RT-PCR

To measure the maximal length of miRNA fragments resistant to GI tract degradation, gene-specific primers and reverse transcriptase were used to convert the pre-miRNA and mature miRNA from stomach and fecal samples to cDNA (Chen et al. 2005; Schmittgen et al. 2008). DNase-treated total RNA (20 *μ*L total volume) was incubated with 1 *μ*L of a cocktail containing 10 mmol/L of each of the antisense primers listed in the table 1. The reagents were heated to 80°C for 5 min to denature the RNA, and the reagents were cooled to room temperature quickly, then the remaining reagents (5 × buffer, dNTPs, DL-Dithiothreitol, RNase inhibitor, primescriptRTase) were added as specified according to the manufacturer's protocol. The reaction proceeded for 30–60 min at 42°C followed by a 5 min incubation at 85°C to inactivate the reverse transcriptase. cDNA was stored for using at −20°C or −80°C.

**Table 1:** Gene-specific primers used to amplify the miRNAs precursors and mature miRNA

Gene	Forward primer (5′→3′)	Reverse primer (5′→3′)
Pre-miR-172a	GCAGCACCATCAAGATTCAC	GCAGCATCATCAAGATTCTCATA
Pre-miR-172b	CAGCACCATTAAGATTCACAT	CAGCATCATCAAGATTCTCATA
RT-miR-172	CTCAACTGGTGTCGTGGAGTCGGCAATTCAGT TGAGATGCAGCA	
miR-172	ACACTCCAGCTGGGAGAATCTTGATGATGC	
RT-miR-824	CTCAACTGGTGTCGTGGAGTCGGCAATTCAGTTGAGTCCCTTCT	
miR-824	ACACTCCAGCTGGGTAGACCATTTGTGAGA	TGGTGTCGTGGAGTC

Real-time quantitative PCR was performed using standard protocols on an Applied Biosystems 7500 Sequence Detection System (Foster, CA). Briefly, 1.25 *μ*L of cDNA was added to 10 *μ*L of 2 × SYBR green PCR master mix (TaKaRa, Dalian, China), 200 nmol/L of each primer, and water to a total volume of 20 *μ*L. The reactions were amplified for 15 sec at 95°C and 1 min at 60°C for 40 cycles. The thermal denaturation protocol was run at the end of the PCR cycles to determine the number of products that were present in the reaction. Reactions were typically run in duplicate. The cycle number at which the reaction crossed an arbitrarily placed threshold (CT) was determined for each gene and the relative amount of each miRNA.

To verify the SYBR Green results and to determine whether plant miR-172 can survive the mouse GI tract, a TaqMan® assay was conducted. TaqMan® probe is the “gold standard” in miRNA detection at present, which can distinguish a one nucleotide difference in sequence and provides a reliable means to assess the survival of miR-172 in the GI tract. The RNA samples from blood, fecal suspensions, and contents of the stomach, intestines, and organs of animals previously fed with 50, 30, or 10 *μ*g of RNA were treated with DNase and followed a similar protocol to that described above in the SYBR Green assay.

### Quantification of percent of plant miR-172 that survives digestion

To determine the percentage of originally administered plant RNA that was present after digestion, the TaqMan probe assay was used. Standard curves were constructed for mature miR-172 using a standard sample and quantifying 10-fold dilutions by real-time PCR. After collecting and quantifying the miR-172 present in the various tissues and samples, percentages of the originally administered RNA were determined.

## Results

### Typical miRNAs are present in plants and mice

**Figure 1:**
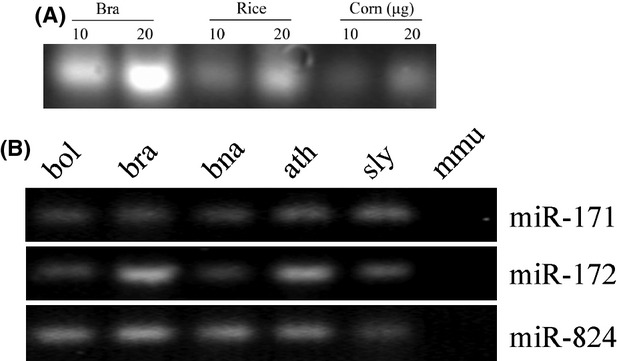
General characterization of small RNAs in different species. (A) Visualization of small RNAs in *Brassica rapa* (bra), rice, and corn. Total RNA from bra tissue, mature dry rice grain, and corn seeds. (B) RT-PCR characterized the expression of miRNA in different species. Upper, middle, lower panel: detection of miR-171, miR-172, and miR-824 in *B. oleracea* (bol), *B. rapa* (bra), *B. napus* (bna), *Arabidopsis thaliana* (ath), *Solanum lycopersicum* (sly), and mouse (mmu). The expression levels of indicated miRNAs evaluated by semiquantitative RT-PCR analysis with 30 cycles.

Total RNA extracts from plants showed a maximal yield of 905.3 *μ*g total RNA per gram of mature plant sample, with 371.6 *μ*g being the average total RNA yield per gram of mature seeds or plant tissue from seven independent RNA extractions (range 214.2–905.3 *μ*g per gram of sample) (Table S1). After electrophoresis, distinct bands ∼20 and 24 nt long (small RNAs) were detected on the agarose gel. Interestingly, small RNAs were only slightly more abundant in developing tissue (*B. rapa*) than in mature seeds (rice, corn) (Fig. 1A). Gel analysis was repeated three times with independent RNA extractions and similar results were obtained.

Selected miRNAs (miR-171, miR-172, and miR-824) showed high levels of expression in *B. oleracea*, *B. rapa*, *A. thaliana*, and *S. lycopersicum* as detected by semiquantitative RT-PCR analysis with 30 cycles (Fig. 1B). However, none of these were detected in fecal samples of control mice, showing the specificity to plants.

### Time course of RNA degradation in animal serum and fecal suspensions

**Figure 2:**
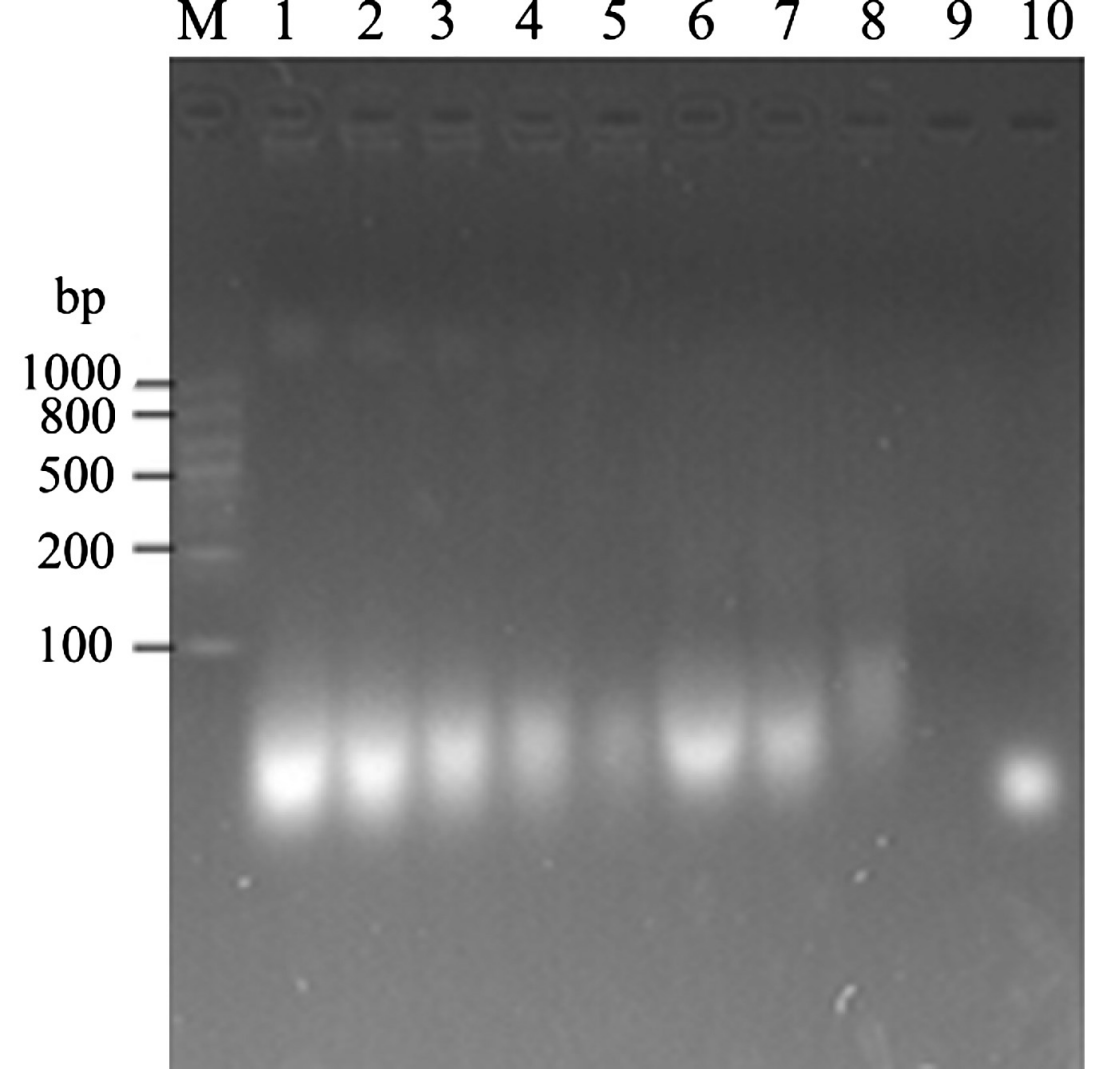
Plant RNA degradation in vitro at different time points. Equivalent amount (5 *μ*g) of RNA was incubated with serum (1–5) or fecal suspension (6–9) at various times. 1, 2 h; 2, 4 h; 3, 10 h; 4, 24 h; 5, 36 h; 6, 2 h; 7, 4 h; 8, 10 h; 9,18 h. M, RNA marker RL1000; 10: synthesized 21nt RNA oligo.

Despite the lack of a low-molecular weight RNA marker, we can infer that the bright bands on the agarose gel are small RNA fragments in the range 20∼100 bp that survived degradation after different times of incubation (Fig. 2). In serum, there were large amounts of RNAs that survived after 24 h of incubation. After 36 h incubation, however, only about 5% of the RNAs were detected. At later times (72 h), the existence of RNA could still be detected (data not shown). However, in fecal suspensions, all RNA molecules were undetectable after 18 h of incubation. These results suggested that small RNA in the serum is more resistant to degradation than in fecal suspensions.

### Presence of Pre-miR-172 and mature miR-172 in the stomach and feces

**Figure 3:**
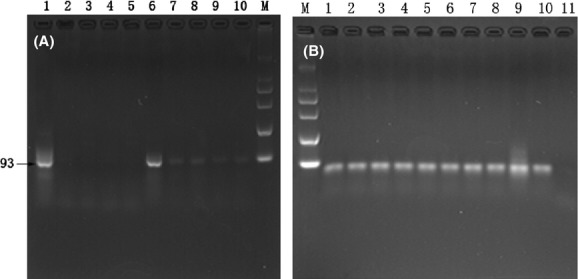
PCR amplification of Pre-172 and miR-172 from the feces of mice at different times after feeding. (A)1, 6: RNA extracted from bra; 2–5: PCR amplification of pre-miR-172a, RNA extracted from stomach, feces of different mice 1 h after feeding. 7–10: PCR amplification of pre-miR-172b, RNA extracted from stomach, feces of different mice 1 h after feeding. (B) PCR amplification of mature miR-172 from the feces and stomach of mice after feeding 2 h. 1–5: fecal samples from different mice; 6–10: from stomach of different mice; 11: sham control.

Since the length of small RNA detected from the gel is in 20–100 bp, selected primers located in the pre-miRNA region were used to measure the maximal length of miRNA fragments resistant to GI tract degradation, and could still be recovered from the feces. The results revealed that pre-miR-172a fragments of ∼100 bp could not be detected, and only a trace amount of pre-miR-172b was detected in the stomach 1 h after feeding (Fig. 3A). In the feces, neither pre-miR-172a nor pre-miR-172b was detected after 1 h incubation (data not shown). Mature miR-172, however, was clearly detected in the contents of the stomach and feces after 2 h incubation (Fig. 3B). It is noted that pre-miR-172 was never detectable in mock-fed animals (received PBS devoid of small RNA) or in the blood from RNA-fed mice 2 h after feeding. Therefore, we focused on mature miR-172 for the remainder of the study.

### The exogenous plant miRNAs in food can pass through the mouse GI tract and enter the blood and organs

We show through RT-PCR that miR-172 is present in the contents of the stomach, intestines, and feces of mice fed total RNA directly by pipette or administered in food. The content of the stomach, intestines, and feces were miR-172-positive between 2 and 72 h, with maximal amounts present from 2 to 4 h after feeding. Pre-miR-172 can no longer be detected 2 h after feeding, however, the miR-172 still can be detected in fecal samples.

Next, the contents of stomach, intestines, blood were investigated, and the results suggested that contents of the stomach and intestines of mice all contained miR-172 at various time points after feeding. However, it should be noted that miR-172 can be detected distinctly in the RNA extracted from whole blood or from the serum of animals at various times after feeding 50 *μ*g of RNA to mice; however, in some experiments, when 30 *μ*g or 10 *μ*g of RNA were fed, the blood of mice had not received miR-172. To determine whether plant miR-172 that survived in mouse GI tract could enter the blood, we used TaqMan® assay method to verify the result of conventional RT-PCR. The results presented in Fig. 5D show that miR-172 is present in the blood of mice between 2 h and 72 h after feeding.

**Figure 4:**
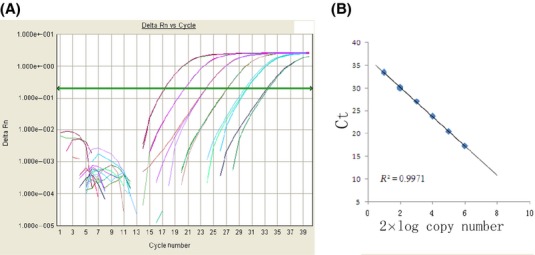
Determination of mature miR-172 copy number. (A) Standard curves were constructed for mature miR-172 using synthetic miR-172 standard sample. Tenfold dilutions of miR-172 RNA oligos was quantified by real-time PCR. (B) The assays produced a 6-log dynamic range, lower limit of detection of 2 × 10 copies and an *R*^2^ > 0.99.

**Figure 5:**
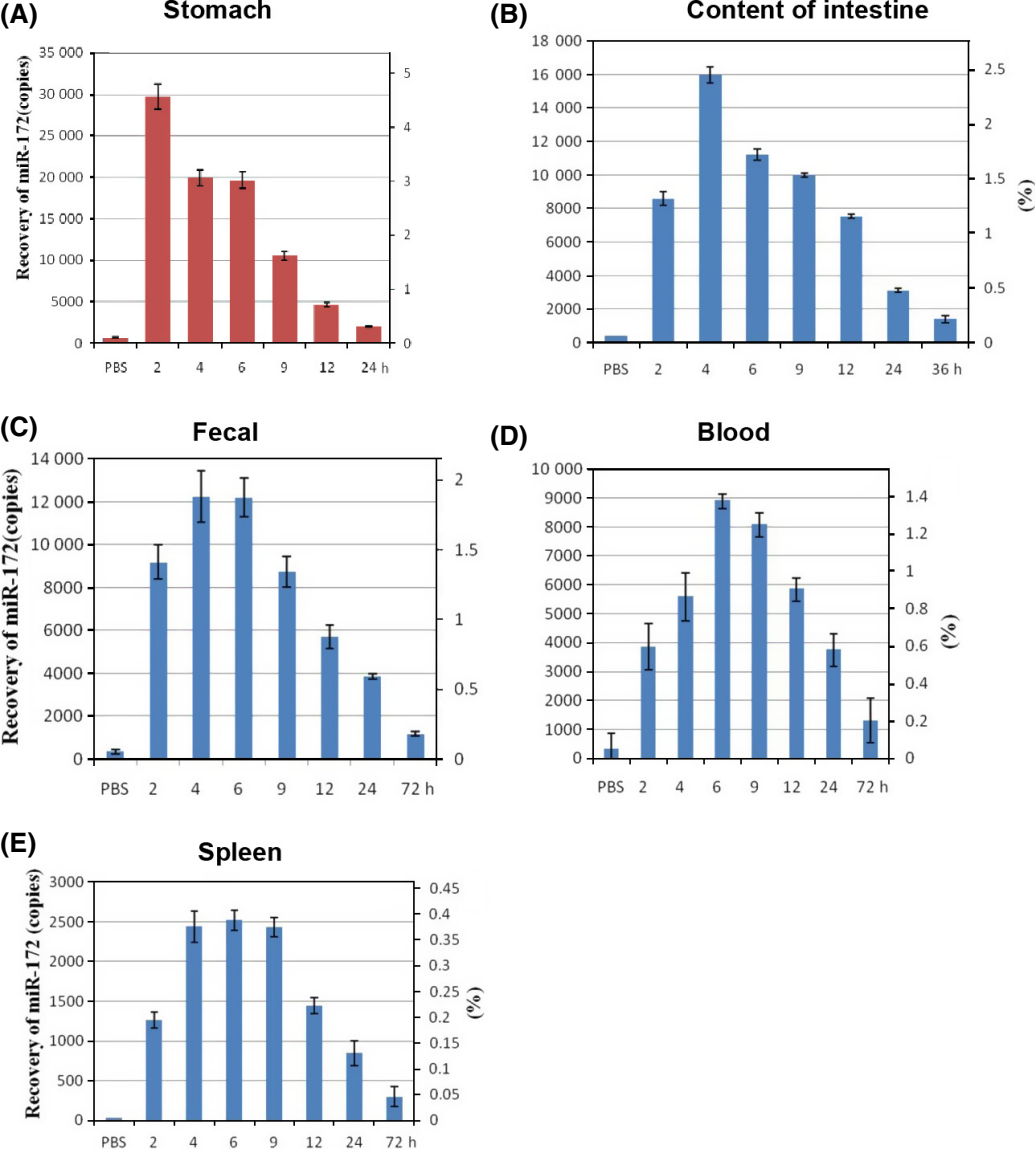
Quantitation of miR-172 foreign RNA detectable by PCR in different segments of the digestive system (A–C), in peripheral blood (D) spleen (E). Fifty micrograms of small RNA (19–100 nt) was given to 2- to 4-month-old mice. As indicated, at times after feeding, the RNA was extracted (1) from the contents of the stomach (A), intestine (B), feces (C), blood (D) or spleen (E). The persisting small RNA was identified by PCR. Considering the fraction of the total re-extracted RNA from gut segments, blood or tissue submitted to the PCR, percentages (scales on right) of the 50 μg of orally administered small RNA were calculated. The scales in approximate copies on the left referred to the actual copies for reference quantitative PCR.

Furthermore, we attempted to quantitate the proportion of the orally administered RNA that survived in the different samples with the standard curve (Fig. 4). The results of a TaqMan probe experiment suggested that of the order of 0.3–1.8% of the miR-172 originally administered could be recovered from the feces (Fig. 5C). By comparison to the results of reconstitution experiments it was determined that the stomach contained about 4.5–0.4% (2–24 h after feeding), the intestines 2.4–0.2% (2–36 h), blood about 1.3–0.2% (2–72 h), and spleen about 0.38–0.04% (2–72 h) of the miR-172 orally administered (Fig. 5). It is essential to note that, in some of the experiments, 30–50 *μ*g of small RNA was added to the food provided to the mice. Obviously, under this feeding regime, the exact time of ingestion of small RNA could not be determined. We hence recorded the times of feces collection after offering the small RNA-treated food. In this case, miR-172 could be detected in the content of the stomach and intestines of mice at 2, 4, 9, 24, until 36 h. after presenting the food containing small RNA, but at 72 h after pellet exposure, the amount of miR-172 was significantly less.

After a single feeding of foreign RNA to mice, this foreign miRNA could be detected by PCR method (other organs) up to 36 h in the contents of the intestine (Fig. 5B), up to 72 h in blood, spleen or excrement. However, miR-172 was merely detectable in kidney and liver (data not shown), and even long after a single feeding was found to be devoid of the small RNAs.

In addition, from the experiments carried out, there was no evidence that differences in the sex of the animals or their age within the 2- to 4-month range affected the outcome of any of the experiments reported (Table S2).

## Discussion

RNA is an unstable molecule that degrades easily under normal environmental conditions (Krutzfeldt et al. 2007; Kallman et al. 2013). However, it was recently reported that some small RNAs can survive extreme environmental conditions in vitro or in vivo (Chen et al. 2008; Mitchell et al. 2008). This begs the question can these RNA also withstand the enzymatic machinery of the GI tract in mammals? The answer to this question has considerable implications, as the digestive tract of all organisms is constantly exposed to many types of foreign DNA/RNA often coated with proteins. The epithelial lining of the GI system could thus represent a primary portal of entry for nucleic acid molecules (Schubbert et al. 1997), provided they survived in the GI tract, at least in the form of fragments. This study determined to what extent foreign nucleic acid can be recovered from the GI tract of mammals, as well as compartments beyond the GI tract, such as the blood and various organs.

In this study, miR-172 was chosen as the focus as we found no identical sequence between *Brassica oleracea* and mammals registered in the miRbase-database. As well, miR-172 was not detected in the total RNA extracted from the mice livers, spleens, or NIH/3T3 cells, or in excrement of mice. First, we studied exogenous RNA degradation in vitro, by measuring plant small RNA after incubating for 2–36 h in blood and fecal samples. The result suggesting that length of 20–100 nt small RNA can survive for 36 h or longer in blood and fecal samples, in the presence of degrading enzymes.

Next, miRNA survival in the GI was measured in mice fed exogenous plant RNA, as well as investigating the presence of miR-172 sequences in liver, spleen, kidney, whole blood, and serum of the animals fed with plant RNA. Between 2 h and 72 h after feeding 50 *μ*g of plant total RNA extracted from Brassica oleracea to mice, fecal samples were positive for miR-172. In a separate experiment, even after feeding as little as 10 *μ*g of total RNA, miRNA-172-specific signals could be detected by PCR (data not shown). Despite the presence of mature miR-172 in fecal samples, pre-miR-172a and pre-miR-172b were not detected in mice feces 2 h after feeding. It is apparent that pre-miR-172 decomposed in 2 h, while miR-172 could survive between 2 and 72 h after feeding RNA. At later times after feeding, pre-miR-172 could no longer be detected in the mice. There was no evidence for long-term survival of pre-miRNA in the intestines of the mice.

These results suggested that miRNA can withstand degradation in the adult mice GI tract and can be detected in the content of stomach and intestines between 2 and 72 h after feeding. Similar results were obtained when 30 *μ*g or 10 *μ*g of RNA were fed to mice (Table S2). The small percentage of dietary RNA detected indicates a low level of uptake, and is consistent with other recent works (Nicklin et al. 1998; Snow et al. 2013).

Generally, the GI tract is the system most likely to take up foreign nucleic acid, and is constantly exposed to the foreign nucleic acid ingested with food. We have directly tested this by feeding a readily available, well-characterized foreign RNA, added to food or by gavage, to mice whose feces was void of this miRNA prior to feeding. After feeding plant total RNA to the mice, their feces were tested for the presence of miRNA. These investigations were then extended to the blood and other organs of the treated animals. We presume that miR-172 behaves similarly to many other miRNAs in the GI tract of mice, but has not yet been tested in humans directly.

All feeding experiments and analysis of the stomach, intestines, feces, and blood performed over the past 2 years in our laboratory are listed in Table S2. Foreign RNA administered to the oral cavity of mice can be detected in the feces, with about 1.8% of the mature miRNA surviving to the feces of animals between 2 and 6 h after feeding. The time course of appearance and disappearance of small RNA in the feces and in the blood of animals reflects the relative stability of some small RNA when incubated with blood or fecal suspension in vitro. The finding that a small percentage of miRNA can be detected in the GI tract suggests that miRNA can survival in the murine GI tract. The presence of miR-172 in the spleen, liver, and kidney suggests that foreign miRNA can exist in the murine body. These findings indicate that the transport of foreign small RNA is likely through the blood stream and into respective organs.

The observation that the foreign small RNA fed to mice can be retrieved from their bloodstream and organs has serious implications. It raises several questions, such as whether this small RNA can be taken up by cells in the different organ systems, and whether the foreign small RNA can be resorbed by the GI, and play functional roles in the animal?

Results obtained with the highly sensitive PCR method have to be critically evaluated for the possibility of unintended, haphazardly occurring contamination with minute traces of the experimental small RNA. Although some weak positive results occurred in a few control experiments (RNA samples from the feces from untreated animals), the relatively large amount of exogenous miRNA detected in test samples reassures us that exogenous plant miRNAs did survive in the GI tract. Moreover, the transient survival of small RNA in the GI tract, feces, blood, and organs have been proved by TaqMan probe methods, and is a method generally accepted.

The amount of foreign RNA administered to mice was between 10 and 50 *μ*g. These values must be related to the body weight of mice (between 15 and 25 g), and thus constitute an approximate fraction of 2/10^6^ of their body weight. For humans of average weights between 50 and 70 kg, one would thus have to postulate an intake of about 50–140 mg of RNA with the daily food uptake to parallel the situation simulated in these mice experiments. Based on the estimate of ∼500 *μ*g of total RNA yielded per gram mature seeds, this would require about 280 g of food consumed, a reasonable human exposure.

The stability of exogenous RNA in organisms and whether it can enter cells and elicit certain functional control in the recipient cells remains unclear. Zhang et al. (2012) reported that orally administered plant miRNA were present in the sera and tissues of animals, and furthermore, found that plant miR-168a can inhibit LDLRAP1 expression in the liver and consequently decrease low density lipoprotein removal from mouse plasma. In contrast, O'Neill et al. (2011) indicated that following oral administration of plant miRNA, the hostile environment of the gut posed significant barriers to stability. Furthermore, Snow et al. (2013) indicate that levels of RNA in cells after ingestion are insufficient for regulation of gene expression, and Petrick et al. (2013) and Witwer et al. (2013) have called into question the claims that ingested miRNAs from rice regulate gene expression in mice.

**Figure 6:**
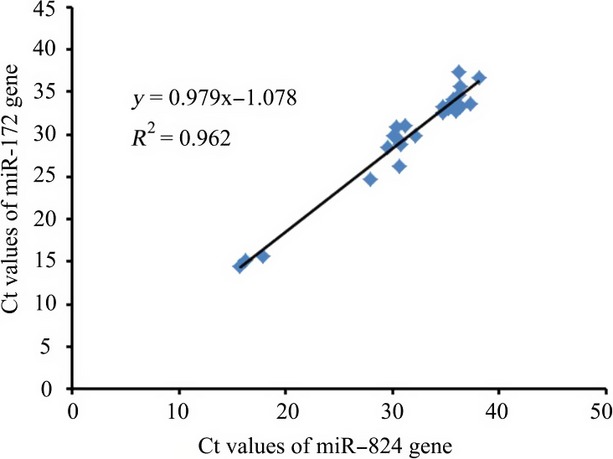
Correlation between recovery of miR-172 and miR-824 in mice excrement.

In this context, many questions remain to be investigated, none of which will be technically easy to control. Our data are consistent with the notion that a steady flow of exogenous genetic material persists in the GI tract, and a very small percent can enter the circulation and distribute in different organs (Zhang et al. 2012). Understanding whether small RNA survival in mammals is specific to miR-172 or similar to other small RNA is of interest. While measuring miR-172, we also tested the presence of miR-824 after feeding 50 *μ*g of RNA to mice, and the result suggests that there is a good correlation between miR-824 and miR-172 (Fig. 6). The present findings, however, must be reproduced with other types of small RNA.

While our results do not support general and consistent uptake of dietary plant miRNAs, additional studies are needed to establish whether or not plant or animal xeno miRs are transferred across the GI in sufficient quantity to regulate endogenous gene expression, and these experiments have been initiated (Witwer et al. 2013).

## Conclusion

This study shows that foreign plant miRNA ingested by mammals can survive through the GI system, and enter the bloodstream and various organs of mice. Experiments involving foreign miRNA entering into established mammalian, and probably other eukaryotic, genomes have to take into account the possibility that these foreign small RNA may have important consequences, with unexpected alterations of mammalian genome and expression patterns. It is important to note that we have not found phenotypic changes in all the mice fed with the foreign RNA.

## Supporting Information

**Table S1.** Yield of total RNA in different materials. **Table S2.** Transient survival of orally administered small RNA in the stomach, intestine, and feces of mice.
