# Supplementary material for: Assessing the survival of exogenous plant microRNA in mice
Source: Food Sci Nutr. 2014 May 15;2(4):380–8. doi: 10.1002/fsn3.113 (PMC4221836; doi:10.1002/fsn3.113)
Supplement: Supplementary file 1 [file fsn30002-0380-SD1.docx]

Supplemental table 1.Yield of total RNA in different materials.

| sample | Bra | Rice | Corn | Bol | Bna | Ath | Sly |
| --- | --- | --- | --- | --- | --- | --- | --- |
| OD_260_/OD_230_ | 1.92 | 1.97 | 2.12 | 1.87 | 2.27 | 2.44 | 2.22 |
| OD_260_/OD_280_ | 1.82 | 1.74 | 1.69 | 2.08 | 1.97 | 2.02 | 1.72 |
| Yield(µg/g) | 232.7 | 324.9 | 286.9 | 905.3 | 364.8 | 272.4 | 214.2 |

Supplemental table 2.Transient survival of orally administered small RNA in the stomach, intestine, and feces of mice.

| Experi-ment  No. | No.of animals | sex | Amount of small RNA fed | Results | | | | | |
| --- | --- | --- | --- | --- | --- | --- | --- | --- | --- |
|  |  |  |  | Content of Stomach | Content of Intestine | Feces extract | Blood | Spleen | Liver |
| 1 | 1 ^a^ | F | 30 ug | + | + | NA | + | NA | NA |
| 2 | 1 ^a^ | F | 50 ug | + | + | NA | + | NA | NA |
| 3 | 2 | F | 2x50 ug | 2+ | 2+ | NA | 2+ | NA | NA |
| 4 | 3 ^a^ | F | 3x30 ug | 3+ | 3+ | NA | 2+/1­- | NA | NA |
| 5 | 2 ^a^ | M | 2x30 ug | 2+ | 2+ | 2+ | 2+ | NA | NA |
| 6 | 4 | 3F/1M | 2x30 ug  2x20ug | 2+  2+ | 2+  2+ | 2+  2+ | 2+  2+ | NA  NA | NA  NA |
| 7 | 6 ^a^ | F | 2x50 ug  2x30 ug  2xPBS | 2+  2+  1+/1- | 2+  2+  2- | 2+  2+  1+/1- | 2+  1+/1-  2- | 2+  1+/1-  NA | 2+  1+/1-  NA |
| 8 | 7 | M | 2x30 ug  2x10 ug  3xPBS | 2+  2+  1+/2- | 2+  2+  1+/2- | 2+  2+  1+/2- | 2+  1+/1-  3- | 2+  1+/1-  3- | 2+  1+/1-  3- |
| 9 | 5 ^a^ | 2F/3M | 2x20 ug  2x10 ug  1xPBS | 2+  2+  1- | 2+  2+  1- | 2+  2+  1- | 2+  1+/1-  1- | 2+  1+/1-  1- | 1+/1-  2-  1- |
| 10 | 10 ^a^ | 4F/6M | 3x30 ug  2x20ug  3x10ug  2xPBS | 3+  NA  NA  2- | 3+  NA  NA  2- | 3+  NA  NA  1+/1- | 2+/1-  1+/1-  2+/1-  2- | 2+/1-  1+/1-  2+/1-  2- | 2+/1-  1+/1-  3-  2- |
| 11 | 6 | F | 2x20 ug  2x10 ug  2xPBS | 2+  2+  2- | 2+  2+  2- | 2+  2+  2- | 2+  1+/1-  2- | 2+  1+/1-  2- | 2+  1+/1-  2- |
| 12 | 11 ^a^ | 7F/4M | 3x20 ug  3x15 ug  2x10ug  3xPBS | NA  NA  NA  2+/1- | NA  NA  NA  2+/1- | NA  NA  NA  2+/1- | 2+/1-  3+  2+  3- | 2+/1-  2+/1-  1+/1-  3- | 2+/1-  1+/2-  2-  3- |
| 13 | 4 | M | 2x15ug  2xPBS | 2+  2- | 2+  1+/1- | 2+  1+/1- | 2+  2- | 2+  2- | 2-  2- |
| Total 47 mice fed small RNA; 15 PBS controls | | | | | | | | | |

These experiments were carried out between 2008 and 2010

^a^ This sample was added to food pellets, in all other cases RNA was administered by pipette feeding,

In PCR experiments, the specific amplified band of expected size also verified by TaMan probe. F, female; M, male; NA, Not analyzed; +, Positive; -, Negative.
